# Comparative Metagenomic Analysis of Soil Microbial Communities across Three Hexachlorocyclohexane Contamination Levels

**DOI:** 10.1371/journal.pone.0046219

**Published:** 2012-09-28

**Authors:** Naseer Sangwan, Pushp Lata, Vatsala Dwivedi, Amit Singh, Neha Niharika, Jasvinder Kaur, Shailly Anand, Jaya Malhotra, Swati Jindal, Aeshna Nigam, Devi Lal, Ankita Dua, Anjali Saxena, Nidhi Garg, Mansi Verma, Jaspreet Kaur, Udita Mukherjee, Jack A. Gilbert, Scot E. Dowd, Rajagopal Raman, Paramjit Khurana, Jitendra P. Khurana, Rup Lal

**Affiliations:** 1 Department of Zoology, University of Delhi, Delhi, India; 2 Argonne National Laboratory, Argonne, Illinois, United States of America; 3 MR DNA (Molecular Research LP), Shallowater, Texas, United States of America; 4 Interdisciplinary Centre for Plant Genomics & Department of Plant Molecular Biology, University of Delhi South Campus, New Delhi, India; 5 Department of Ecology and Evolution, University of Chicago, Chicago, Illinois, United States of America; Washington State University, United States of America

## Abstract

This paper presents the characterization of the microbial community responsible for the *in-situ* bioremediation of hexachlorocyclohexane (HCH). Microbial community structure and function was analyzed using 16S rRNA amplicon and shotgun metagenomic sequencing methods for three sets of soil samples. The three samples were collected from a HCH-dumpsite (450 mg HCH/g soil) and comprised of a HCH/soil ratio of 0.45, 0.0007, and 0.00003, respectively. Certain bacterial; (*Chromohalobacter*, *Marinimicrobium, Idiomarina, Salinosphaera, Halomonas*, *Sphingopyxis, Novosphingobium, Sphingomonas* and *Pseudomonas*), archaeal; (*Halobacterium, Haloarcula* and *Halorhabdus*) and fungal (*Fusarium*) genera were found to be more abundant in the soil sample from the HCH-dumpsite. Consistent with the phylogenetic shift, the dumpsite also exhibited a relatively higher abundance of genes coding for chemotaxis/motility, chloroaromatic and HCH degradation (*lin* genes). Reassembly of a draft pangenome of *Chromohalobacter salaxigenes* sp. (∼8X coverage) and 3 plasmids (pISP3, pISP4 and pLB1; 13X coverage) containing *lin* genes/clusters also provides an evidence for the horizontal transfer of HCH catabolism genes.

## Introduction

From the early 1950 s to late 1980 s, hexachlorocyclohexane (HCH) was one of the most globally popular pesticides used for agricultural crops. HCH is chemically synthesized by the process of photochlorination of benzene. The synthesized product is called as technical-HCH (t-HCH) and consists of five isomers namely, α- (60–70%), γ- (12–16%), β- (10–12%), δ- (6–10%) and ε- (3–4%) [1]. The insecticidal property of HCH is contributed mainly by the γ-HCH (also known as Lindane) [2]. The process of extracting the γ -HCH isomer from the t-HCH generates a HCH-waste (consisting of α-, β-, δ- HCH) which is 8 times the amount of lindane produced [1]. In the last 60 years, 600,000 tons of lindane has been produced, thereby, generating a HCH-waste (referred as HCH-muck) of around 4–7 million tons [3]–[4]. The inappropriate waste-disposal techniques and the indiscriminate use of this pesticide have created a global environmental contamination issue [1].

This environmental contamination is mainly associated with the physicochemical properties of the HCH isomers which are completely different from other pollutants [5]. The axial and equatorial position of the chlorine atoms around the cyclohexane ring governs the persistence of these HCH isomers in the environment. Over the years, the build-up of huge stockpiles of HCH waste and their leaching into the environment through air and water have marked HCH as a problematic polluting compound [3]. A primary concern is the human health risks associated with the carcinogenic [6], endocrine disruptor and neurotoxic [6] properties of the HCH isomers. In May 2008 signatories of the Stockholm Convention listed α-, β- and γ- HCH amongst the recognized persistent organic pollutants (UNEP 2009).

Sites heavily contaminated with HCH have been reported from Germany, Japan, Spain, The Netherlands, Portugal, Greece, Canada, the United States, Eastern Europe, South Africa and India [5]. By the 1970’s and 1980’s the usage and production of t-HCH and lindane was banned in most of the industrialized countries. In India the use of t-HCH was introduced in 1950’s and has continued till 1997. However, even after 1997, there remained restricted production and use of lindane [7]. In the last 15 years 7000–8000 tons of lindane has been manufactured and the corresponding HCH-muck been improperly disposed off at several locations [2] (called HCH dumpsites). These HCH-dumpsites form the ideal experimental sites to understand how microbial communities respond to HCH pollution.

Owing to the global presence of the HCH open sinks, several primal efforts have focused on developing an efficient bioremediation technology [8]–[11]. As a first step the genetics, biochemistry and physiology of microbial degradation of HCH isomers especially of γ-HCH has been studied in detail in Sphingomonads. For example, the genetic pathways responsible for the degradation of γ HCH, also called *lin* genes (*lin* pathway), have been characterized from *Sphingobium japonicum* UT26 [12] and *Sphingobium indicum* B90A [5], [13]. In general γ-HCH degradation pathway is divided into upper and lower pathways. The upper pathway of γ-HCH is mediated by dehydrochlorination (*linA*), haloalkane dehalogenation (*linB*) and dehydrogenation (*linC/linX*) in a sequential manner leading to the formation of 2, 5-dichlorohydroquinone. 2, 5-dichlorohydroquinone (lower pathway) is further converted to succinyl-CoA and acetyl-CoA by the action of reductive dechlorinase (*linD*), ring cleavage oxygenase (*linE*), maleylacetatereductase (*linF*), an acyl-CoA transferase (*linG, H*) and a thiolase (*linJ*). By and large the expression of *lin* genes in these strains is heterogeneous in nature as the genes of the upper pathway are expressed constitutively (*linA, linB* and *linC*) [14] and others (*linE* and *linD*) can be induced *via* transcription factors (*linR*) [14]–[15]. In addition to their primary role in the degradation of γ-HCH, *linA* and *linB* play an important role in the degradation of α-, β-, δ- and ε-HCH; but they also degrade the intermediates that are constitutively generated by this pathway. Sequence differences in the primary LinA and LinB enzymes in the pathway play a key role in determining their ability to degrade the different isomers. These studies formed the base of field trials where *Sphingobium indicum* B90A has been used as a primary bioremediatory element; however, these efforts have had limited success [9]. While one organism may play a dominant role in the degradation process, the role of the associated microbial species in the microbial consortia may also play a role in augmenting its capability. Therefore, characterizing the microbial community structure at HCH-dumpsites should be a priority.

Here we present results of the first detailed investigation of the unexplored bacterial, archaeal and fungal diversity that exists in the soil of a HCH dumpsite. In addition to the taxonomic characterization, changes in their functional dynamics are also studied. The comparative gene centric analysis performed in this study clearly indicate that the marked differences in the microbial community are associated with the changes in the functional diversity especially related to their membrane transport, chemotaxis/motility and catabolic genes (*lin* genes) affected by the presence of HCH isomers at the dumpsite.

## Materials and Methods

### Ethics Statement

No specific permits were required for the described field studies.

### Selection of HCH Contamination, Soil Sampling and Total DNA Extraction

To study the shift in microbial community structure across the increasing HCH contamination, we collected bulk soil samples from a HCH dumpsite situated at Ummari village, Lucknow [7] (27° 00′ 24.7′′ N, 81° 08′ 57.8′′ E), along with two more locations situated at a distance of 1 km (27° 00′ 31.1′′ N, 81° 08′ 54.7 E) and 5 km (27° 00′ 59.5′′ N, 81° 08′ 36.0.8 E) away from the dumpsite. The latter two soils were used as reference to assess the changes in microbial community under HCH stress at the dumpsite. Sampling was performed in the September of 2010 considering seasonal crop rotation (land was not processed for farming). Since sampling sites represent physicochemically different soils from uncultivable (HCH-dumpsite, 450 mg/g) to agriculturally managed (a small segment at 5 km site), subsamples (10 subsamples from each composite mix; 500 g soil/subsample) were collected at a depth of 10–20 cm, coordinates with any type of vegetation (natural or agricultural) were strictly avoided. Sub-samples were transported on ice (4°C) and stored at −80°C till processed for HCH residue estimation and physiochemical analysis using methods described earlier [7]. DNA from each subsample was isolated by using PowerMax® Soil DNA Isolation Kit (MO-BIO, USA). Equal concentration ( = 200 µg) of environment DNA from each subsample (10 subsamples/composite pool) were mixed to form a composite genetic pool representing total DNA composition for each site. DNA purity and concentration was analyzed by using NanoDrop spectrophotometer (NanoDrop Technologies Inc., Wilmington, DE, USA). Isolated total DNA was stored at −20°C till processed for microbial diversity and sequence analyses.

### Sequence Data Generation

We performed targeted amplicon and shotgun pyrosequencing of the environment DNA using titanium protocols (Roche, Indianapolis, IN, USA). Roche 454 analysis software version 2.0 was used to analyze the sequences. The Tag-Encoded FLX Amplicon Pyrosequencing (TEFAP) was performed as described earlier [16] by using one-step PCR, mixture of Hot Start and HotStar high fidelity Taq polymerases. For shotgun sequencing of environmental DNA samples a full picotitre plate was run for each shotgun pyrosequencing library representing individual soil gradient. A total of 1.2 Gigabases of nucleotide sequence was generated (Table 1). Raw reads were processed for various quality measures using Seq-trim pipeline [17]. Reads were preprocessed at the following parameters; minimum length = 250 bp, minimum quality score = Phred Q20 average and reads with ambiguous bases (including N) were not used for further analysis.

**Table 1:** Chemical properties and sequencing data of soil gradients with HCH gradient.

Characteristic	Dumpsite	1 Km	5 Km
pH	7.21	7.81	7.93
EC (dS/m)	8.50	0.19	0.43
Organic carbon (%)	30.74	0.45	0.67
Available K (kg/ha)	918	40.5	84.3
Available P (kg/ha)	60.3	93	318
Available N (kg/ha)	335	397	460
Salinity	Highly Saline	Normal	Normal
∑HCH (mg/g)	450	0.7	0.03
Sequence count	1,187,505	1,124,891	1,187,505
Mean length bp (SD)	337 (112)	339 (126)	337 (112)
Mean GC % (SD)	62 (9)	60 (10)	62 (9)

∑HCH: represents the sum of α and β HCH isomers concentration.

Salinity levels are representing the EC and cation concentration.

### Microbial Diversity Analysis

We estimated microbial diversity across increasing HCH contamination by using three different methods: TEFAP, metagenomic SSU rRNA typing and direct comparison of EGTs (Environmental Gene Tags) to the reference genomes. For bacterial, archaeal and fungal diversity analysis by TEFAP [16] method a total of 6 individual primer sets were utilized (Table S1). Following sequencing, all failed sequence reads, low quality sequence ends (Phred Q20 average) and tags/primers and reads <250 bp were removed. The resulting sequences were then deleted of any non-bacterial/archaeal/fungal ribosome sequences and chimeras using custom software [18] set at default parameters. For archaeal analysis, in addition to the above steps, sequences with greater identity to bacterial 16S rRNA gene sequences were also deleted. Unique reads were BLASTN [19] (E-value cutoff of 1×10^−5^ minimum coverage 90% and 88% identity) against GreenGene [20] (16S rRNA) and SILVA [21] (SSUs and LSUs) databases. Resulting outputs were compiled and data reduction analysis performed by using a NET and C# analysis pipeline [22]. In the second approach SSU rRNAs from the shotgun metagenomic sequences were binned from each metagenome using BLASTN [19] (E-value cutoff of 1×10^−10^ minimum coverage 90% and 88% identity) against rRNA databases mentioned above. OTU (Operational Taxonomic Unit: status was assigned to sequences above 300 bp and similar to reference sequences (>95%). OTUs were clustered with 97% similarity criteria using UCLUST [23]. Candidates OTUs were used to assign phylogeny using RDP [24] scheme at 80% confidence value [25]. Relative abundance matrix (genus) of the metagenomes was used for statistical analysis. In the third approach taxonomical profiles were constructed by mapping metagenomic reads against NCBI genome database using NBC [26] (Naive Bayesian Classifier) at a N-mer length of 12.

### Qualitative and Quantitative Measurements of Phylogenetic Diversity

For each metagenome, a subset of 1000 randomly selected candidate OTUs were used to construct a relaxed neighbor-joining tree using Clearcut [27] with Kimura correction. To understand the phylogenetic correlation between sampled soil cohorts, distance matrices were constructed from each phylogeny and Mantel test (10000 permutations, two tailed: p-value) was performed using PASSAGE-2 [28]. Additionally, un-weighted UniFrac [29] was run on phylogenetic tree (at 1000 permutation) constructed after combining candidate OTUs from each metagenome. Rarefaction plots and non-parametric diversity indices were calculated using EstimateS [30]. The statistics utilized are not based upon biological replications but instead based upon technical replications provided by utilizing multiple diversity assays. Thus, we are representing the observational evaluation of the 3 samples analyzed using a variety of diversity assays and metagenome sequencing data from samples with three different contamination levels of HCH.

### Characterization of Metagenomic Gene Content

Metagenomic sequences were annotated using evidence based annotation approach [31]. Sequences were BLASTX [19] against several protein databases (COGs, Pfam, SWISS PROT/TREMBLE and KEGG) at an E-value cutoff: 1×10^−5^. Predicted genes were tabulated and classified into functional categories from lower orders (individual genes) to higher orders (cellular processes). Relative abundance for each gene was calculated by dividing the similarity hits for an individual gene by total hits against any of the database. Higher functional order enriched in any of the metagenome was later analyzed at the finer scales. To understand the gradient specific functional traits, endemic metagenomic reads were binned using MegaBLAST [32] (reads of one metagenome against combination of remaining).

### Community Potential and Participation for HCH Degradation

Sequences for well-characterized HCH degrading genes (Table S8) were downloaded from NCBI (dated 11^th^ March, 2011) and utilized as a template for DNA-Seq based analysis that was performed using ArrayStar (DNAstar) at default settings. Relative expression was calculated in each metagenome as per manufacturer’s guidelines followed by statistical analysis (Two sided Fishers exact test and storey’s FDR method). Additionally, metagenomic reads representing any of the *lin* gene were binned (BLASTN at E-value; 10^−10^ and 85% query coverage), and reference assembled on the ORF of respective *lin* gene. As mentioned above, protein guided DNA assembly for each *lin* gene was performed using Transpipe [33]. Relative abundance of lindane degradation pathway was quantified for each HCH gradient *via* comparing extracted *lin* gene sequences against KEGG [34].

### Microdiversity Analysis of the Environmental Genomes

**Figure 1:**
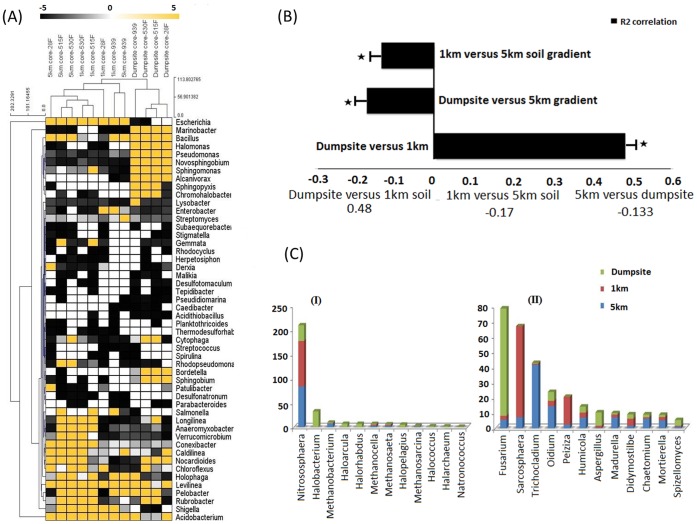
Phylogenetic analysis of the microbiomes. (A) Dual dendrogram of top 50 bacterial genera across three metagenomes obtained after TEFAP analysis using four bacterial primer sets. Genera and sample categories were clustered using Manhattan distance metric, top 50 genera with standard deviation >0.4 and having at least 0.8% of the total abundance were selected. Colour scale is representing the relative abundance of sequence reads (normalized by sample-mean). (B) Phylogenetic correlation of microbial communities across increasing HCH contamination, a subset of 1000 randomly selected OTUs from each metagenome was used to construct an elucidan distance matrix. Matrices were pair-wise compared using Mantel-test (1000 permutation, 0.05 as standard *P* -value) and Pearson correlation values were calculated. Asterisks indicate the statistical significance *P*<0.001(mean±sm). (C) Relative percentage of reads assigned to different archeal (I) and fungal (II) genera in TEFAP analysis.

Phylogenetic reports created by 16S-rRNA pyro-tag, metagenomic SSUs and EGTs comparison with known genomes revealed the enrichment of genera like *Marinobacter*, *Chromohalobacter, Sphingomonas, Sphingopyxsis* and *Novosphingobium* (Fig.1(A) and Fig.S1) along with increasing HCH contamination. Since most of these genera are genetically and functionally selected to degrade or tolerate HCH [5], we further focused our assembly efforts to assess their genomic and plasmid microdiversity. All metagenomic reads were aligned against the reference genomes (Table S9) and plasmids and recruitment plots were generated using MUMMER [35] as explained earlier [36]. Metagenomic reads were assembled into contigs using velvet_0.5.01 [37] (*k*-mer length = 31). Contigs were BLASTX [19] (E-value = 10^−5^) against NCBI nr (non redundant) database. Phylogenetic identity was given to the contigs using MEGAN [38] at default parameters. Largest clusters were grown by recruiting singlets using Scarf algorithm [39] at following parameters, -g x –x T –c T –l 6–M T -n 2. Coverage was calculated for each contig via aligning metagenomic reads back to the contigs using Mosiak aligner (www.bioinformatics.bc.edu) at default parameters. Reference genome sequences (Table S9) were shredded into 3 kb long pseudo-contigs and concatenated with metgenomic contigs. Pooled contigs (reference genomes and metagenome) were later clustered based upon their tetra nucleotide frequency correlations as explained previously [40]. After performing the length distribution of contig pool following parameters were optimized for tetra-ESOM analysis; minimum length of contig = 1800 bp and maximum size window = 3500 bp. To maximize the use of data contigs were further binned using the %GC character as %G+C varies between species but remain highly constant within species [41]. Contigs were submitted to RAST [42] server for gene calling and annotation.

### Statistical Analysis

Identification of genes or subsystems enriched between any two metagenomes was done using two-sided Fishers exact test with storey’s FDR method for multiple test correction using STAMP [43]. Genes or subsystems were considered as enriched if the p-value was significant along with pair wise comparison of metagenomes. A principle component analysis on correlation matrix with 1000 bootstrap value was performed to compare taxonomic profiles generated after 454 pyro-tagging of 16S-rRNA gene, metagenomic SSU-rRNA typing and direct comparison of EGTs with reference genomes. Two-way clustering was also performed on normalized genus versus metagenome sample (relative abundance from each taxonomy predictions method) matrix with some changes in parameters as methods explained elsewhere [44].

### Data Availability

The TEFAP data were submitted to NCBI SRA under accessions SRA045821.1, SRP008135.1, 260594.1 and runs under SRR342413.1 whereas shotgun sequencing data runs under SRX0964712. Data were also uploaded to MG-RAST [45] (accessions: Dumpsite = 4461840.3, 1 km = 4461013.3 and 5 km = 4461011.3).

## Results and Discussion

### Physicochemical Analysis of Soils

The physicochemical analysis of the composite soil samples from three locations (Table 1) showed significant differences (*P*<0.00001 in all corresponding comparisons; Fisher’s Exact test and Storey’s FDR method) in electrical conductivity (maximum at the dumpsite; 8.5 dS/m). The dumpsite soil sample was highly saline (Electrical conductivity and cation concentration) and available potassium was >10 times higher (918 kg/ha) as compared to other composite samples (1 km = 40 kg/ha, 5 km = 84.3 kg/ha). This difference in electrical conductivity (EC) could be due to higher abundance of ions (especially cations) as a result of pesticide contamination [46] and high potassium concentration is a characteristic feature of soil ecosystems with inherent bioremediation potential [47]. HCH contamination was mainly composed of α- and β- HCH (∑ HCH) and was up to 450 mg/g, 0.7 mg/g, 0.03 mg/g soil from the dumpsite, 1 km and 5 km away soil samples, respectively (Table 1). The levels of ∑HCH reported from the dumpsite are the highest reported from any of the dumpsites studied so far [48]–[49].

### Microbial Diversity Estimation

In our first taxonomic approach we performed 16S rRNA amplicon pyrosequencing (TEFAP, Tag-Encoded FLX Amplicon Pyrosequencing) for each composite genetic pool using kingdom specific primers (Table S1) targeted at the conserved domains of the rRNA genes [16]. Fig. 1 provides an overview of bacterial, archaeal and fungal diversity based on TEFAP analysis. In this analysis a total of 114, 771 sequences with an average length of 338 nucleotides were generated, of which 13,437 and 17,293 were derived from archaeal and fungal assays, respectively. After quality control steps (average quality score = Phred Q20 and tags, primers and reads <250 bp length were removed) a total of 72,178, 4,535 and 14,294 sequences were utilized for bacterial, archaeal and fungal diversity analysis, respectively.

### Bacterial Diversity Analysis

Bacterial diversity was analyzed among the 3 sites using 4 bacterial primer pairs (Fig. 1A). The dual dendrogram is clustered based upon weighted pair average and Manhattan distances. Dumpsite assays were clustered together regardless of which primer was utilized. Two of the primer pairs (530F-1100R and 515F-860R) (Table S1) always demonstrated high similarity to each other independently of the environment analyzed (Fig. 1A), which is to be expected, as they cover a similar region of the 16S rRNA gene, but this also suggests that they retrieve a similar community profile despite potential primer bias.

Several genera demonstrated notable differences (average and standard deviation after each individual assay) between the sites (Fig. 1A). *Pseudomonas* (2.9% ±1.9), *Sphingomonas* (2.8% ±3.2), *Novosphingobium* (2.7% ±1.8), *Sphingopyxis* (1.8% ±2.4), *Marinobacter* (14.8% ±3.1) *Chromohalobacter* (2.7% ±5.6), *Halomonas* (4.4% ±1.1) and *Alcanivorus* (4.2% ±6.1) were more abundant in the dumpsite dataset. The first four of these genera have already been reported to degrade HCH isomers in pure cultures [5]. Interestingly, the dumpsite soil dataset was also found to be enriched for anaerobes *Clostridium* and *Dehalobacter* (Table S2) that are also reported to degrade HCH isomers [50]–[51]. In contrast, the 1 km and 5 km datasets were predominated by *Escherichia/Shigella* (37.8/7.6% ±3.1); *Acidobacterium* (17.3% ±2.6), *Salmonella* (7.6% ±2.3), *Levilinea* (3.5% ±0.7) *and Rubrobacterin* (3.3% ±1.3), respectively. This finding is not unexpected as these bacteria especially *Escherichia/Shigella* commonly colonize soils impacted by human or animal waste, and a small segment of these sites were using such waste as a fertilizer for growing rice, wheat and vegetables.

We also observed bacterial genera which were unique to the dumpsite dataset. The criteria for selection of these genera required that each of the bacterial diversity assays agreed (*i.e.* for the genera all four were positive at the dumpsite and negative at the other sites). These genera and the average percentage (average among the four bacterial diversity assays) are presented in Table S3. *Marinimicrobium* (1.1% ±0.45), *Idiomarina* (0.67% ±0.16) and *Salinisphaera* (0.46% ±0.20) were abundant as well as unique to the dumpsite dataset alone (Table S3). However, there is no clear evidence of their association with the degradation of HCH isomers, nor any documented presence at HCH dumpsites in the literature, although they have been reported from hyper saline environments [52], which suggests that the salinity of the dumpsite could be promoting unique microbial composition. Some of the major genera that were predominantly present at lowest HCH site (5 km) include *Cladilinea*, *Streptomyces* and *Gemmatimonas* (Table S4).

The bacterial/phylum distribution based upon SSU rRNA analysis using RDP [24] (Table S5) was by and large in agreement with that of TEFAP analysis. The most abundant phyla present in the dumpsite and 1 km datasets were Proteobacteria (50–50.8%) followed by Firmicutes (33.8–43%) and Actinobacteria (4–14.5%). In contrast Firmicutes (70%) were most abundant in the 5 km (lowest HCH) dataset (Table S5), which are known to be dominant in dry/arid soils [53]. Fusobacteria, Cyanobacteria and Chlorobi were completely absent in the dumpsite and 1 km datasets. Therefore, while HCH contamination did impact the diversity and abundance of the various bacterial genera, it did not markedly affect phylum level diversity or abundance. A Mantel test of beta-diversity between sites (between distance matrices generated from phylogenetic tree of candidate OTUs; Fig. 1B) indicates a significant linear correlation (*P*<0.001) between increasing stress conditions (HCH contamination and salinity) and microbial community structure. These beta-diversity patterns are driven by the change in diversity and abundance of genera as described above rather than higher taxonomic ranks.

Further insights into the bacterial diversity within the three metagenomic datasets was obtained by computationally identifying the reads matching bacterial 16S rRNA gene sequences from the metagenomic reads (EGTs) and assigning them to different taxonomic levels (SSU rRNA). We also mapped EGTs to >1100 bacterial genomes (EGT genome typing) in NCBI reference genome database [54]. A total of 2,926, 4,164 and 2,301 SSU rRNA reads were obtained from the dumpsite, 1 km and 5 km datasets, respectively. The phylogenetic composition obtained by TEFAP, SSU rRNA and EGT typing analysis was compared at the genus (Fig S1) and phylum level (Fig S2). Despite the general accordance, there are some noteworthy differences between the TEFAP, SSU rRNA typing and EGT typing. For example, *Streptococcus* was more abundant (9.6%) at the dumpsite according to EGT typing in comparison to TEFAP (1%, ±1.2) prediction, while *Acidobacterium* was predominant in TEFAP analysis at the dumpsite (13.3%, ±2.3) in comparison to SSU rRNA typing (1%). Relative enrichment of *Pseudomonas* (*P*<0.001 in all corresponding comparisons), *Sphingomonas* (*P*<0.001 in all corresponding comparisons) and *Chromohalobacter* (*P*<0.001 in all corresponding comparisons) was validated by all three approaches used. Some of the differences among these three techniques could possibly be attributed to the inherent biases of each technique, such as low coverage of 16S rRNA in metagenomic data (SSU rRNA), PCR primer amplification (TEFAP), and lack of relevant genomes for this environment (EGT genome typing) as reported previously [55]–[56]. Two strong points emerge from the data. First, the data reflect that at the surface soil (up to 20 cm) there is relative enrichment of bacterial, archaeal and fungal taxa genetically evolved to tolerate high salinity and degrade HCH isomers. Thus natural attenuation, a process in which microbial community contribute to the pollutant degradation is already in operation but needs to be monitored in detail over several other parameters (salinity, organic wastes and time). Second, for rapid degradation of HCH isomers at the dumpsite, the metagenomic data suggests that it may indeed be possible to effectively biostimulate the indigenous bacterial community by application of specific nutrients that would target the productivity of specific taxa [10]–[11] (taxa specific minimal salt medium and electron donors).

### Archaeal and Fungal Diversity

So far the available literature on microbial diversity at the HCH dumpsites only reflects the presence of bacteria [10], [48]–[49], with archaeal and fungal diversity having never been analyzed at a HCH dumpsite. Based upon relative abundance (reads assigned to a particular archaeal genus/total reads assigned to the archaeal domain), *Nitrososphaera* (>90%) and related genera were enriched in the 1 km and the 5 km datasets whereas in the dumpsite dataset there was a relative increase in the abundance of genera like *Halobacterium* (>30%), *Haloarcula* (>10%), *Halorhabdus* (>10%) and *Halopelagius* (>5%) (Fig. 1C–I). Archaeal genera like *Halorhabdus* [57] and *Halobacterium* [58] have already been reported as naturally selected inhabitants of highly saline (EC and cations concentration) environments. In general, halophilic bacteria and archaea have a broad catabolic potential [52], and hence these halophiles may have a role in HCH degradation at the dumpsite. Evaluation of fungal diversity based upon TEFAP analysis at the dumpsite revealed high proportion of *Fusarium* species (>50%) that were absent in our sampled genetically pooled samples representing two remote sites (Fig. 1C–II). Fusarium species were tentatively identified as either *F. equiseti* or *F. oxysporum* (LSU with >97% sequence similarity to the reference sequence; Fig. S3). While the role of other dominant fungal species is not yet known, the ability of *Fusarium* sp. to degrade HCH isomers in pure cultures has been described previously [59]–[60]. The 1 km site, a certain segment of which is potentially impacted by human or animal waste fertilizer, showed comparatively high proportions of *Sarcosphaera* (48.13%) and *Peziza* (14.67%), while the most distant site (5 km) was relatively high in *Trichocladium* (28.94%) and *Oidium* (10.13%). Unlike the bacterial analysis, there were too few archaeal or fungal sequences identified by rRNA classification or genomic mapping from the metagenomic data to providing meaningful results. Nevertheless the microbial community at the dumpsite and 1 km datasets were more closely related to each other than 1 km-5 km or dumpsite-5 km datasets (Fig. 1A, S1 and S2), validating the HCH contamination and salinity hypothesis. Further increase in sequencing depth and replicates could help to improve the resolution of these findings.

### Metagenome Functional Overview

**Figure 2:**
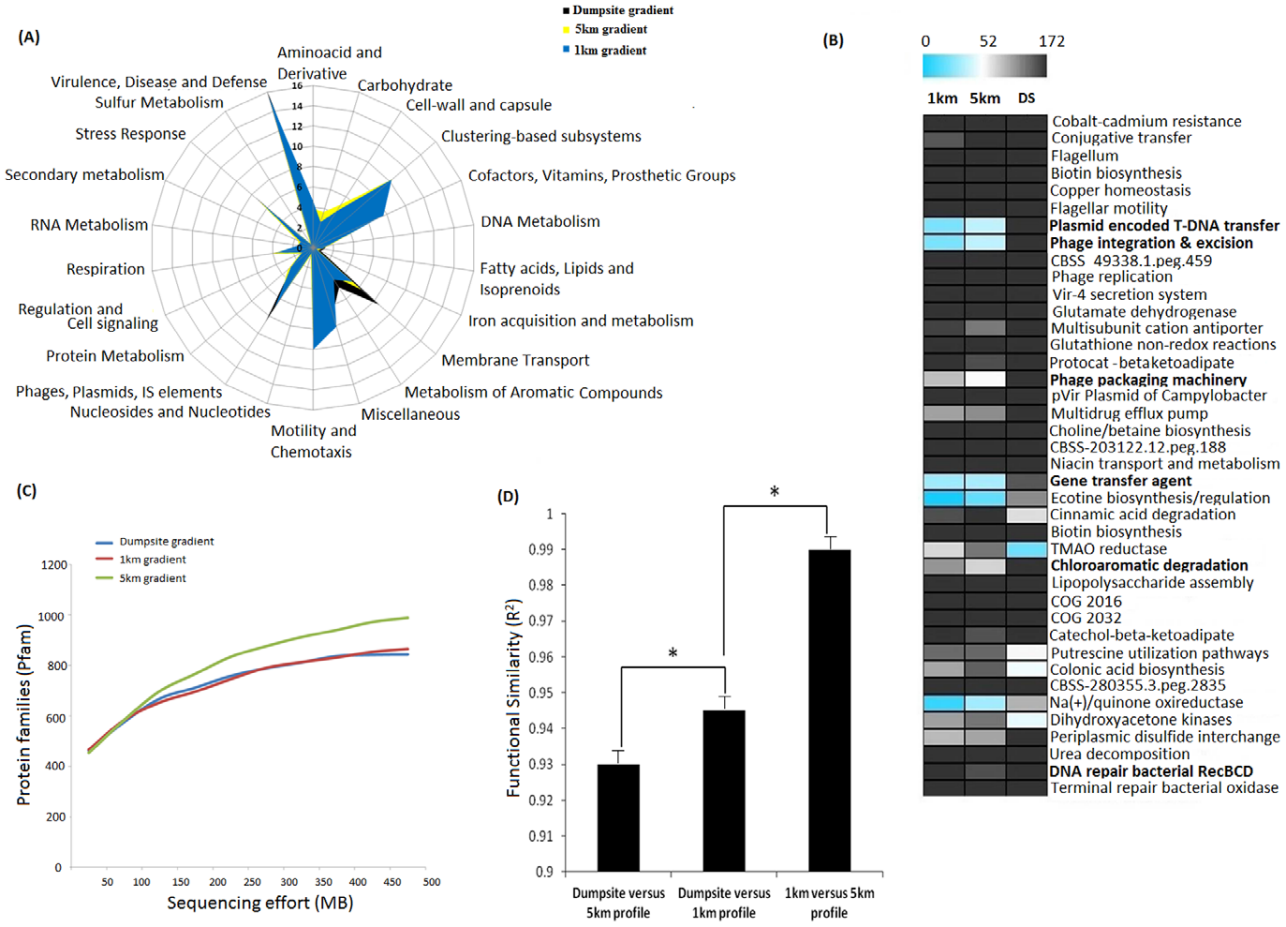
Functional traits of the studied metagenomes. (A) Cellular processes enriched over increasing HCH contamination. Metagenomic reads were compared against the COG database and relative percentage (y-axis) for each category (x-axis) was calculated. (B) Heat map showing the relative abundance of top 50 subsystems enriched over increasing HCH concentrations (percentage cut-off = 0.8%, standard deviation cut-off = 0.4%). (C) Rarefaction analysis performed on unique protein families (Pfam) sampled across three HCH gradients. (D) Comparison of functional categories similarity between metagenome gradient pairs. KEGG enzyme profile of each metagenome was compared. Asterisks indicate significant differences (Two sided Fishers exact test with Bonferroni multiple test correction, *P*<0.01). ABBREVATIONS: (1) **DS** = dumpsite gradient, **1** **km** = 1 km gradient and **5** **km** = 5 km gradient.

Protein functions generated from evidence-based annotation (Pfam, COGs, SWISS PROT/TREMBLE and KEGG databases) were classified at various hierarchies [31] (individual genes, protein families and cellular processes). Observed increase in HCH contamination resulted in an increase in the relative abundance of cellular processes such as membrane transport (*P*<0.001 for all pair wise comparison), motility and chemotaxis (*P*<0.001 for 5 km versus 1 km and <0.01 for dumpsite versus 1 km dataset comparison), transposases and plasmid maintenance (*P*<0.001 for all pair wise comparisons) (Fig. 2A). Additionally, phage and prophage elements were also heightened in the HCH dumpsite, suggesting an increase in genetic mobility due to pollution or salinity stress. Enriched subsystems and protein families involved in each of the above-mentioned processes were identified and characterized (Fig. 2B and Table S6). Categories involved in aromatic compound metabolism include chlorobenzoate, benzoate and toluene degradation (Table S6), which have been reported as end products of anaerobic degradation of HCH [61], were found to be positively correlated to the HCH contamination. Rarefaction estimates (Fig. 2C), two sided Fisher’s Exact test and Storey’s FDR method were performed on the Pfam [62] database results (protein families) using STAMP [43]. Protein families that were significantly higher in the dumpsite include transposons (*P*<1e^−11^ for each pair wise comparisons), phages (*P*<1e^−15^ for each pair wise comparisons), IS elements (*P*<1e^−10^ for each pair wise comparisons), alpha-beta hydrolase folds (*P*<1e^−15^ for each pair wise comparisons), major facilitator super family (*P*<1e^−15^ for each pair wise comparisons) and short chain dehydrogenases (*P*<1e^−15^ for each pair wise comparisons). It is not surprising that an increase in salinity levels and HCH contamination resulted in an increase in the enrichment of microbial genes coding for enzymes and proteins involved in aromatic compound metabolism, stress tolerance, multidrug resistance and motility/chemotaxis proteins. Similarly, the genes involved in motility, chemotaxis and sensing, were required for sensing HCH isomers [63].

Based on SOM (Self Organization Mapping) analysis we observed that genes coding for phage DNA synthesis, capsid proteins, packaging and transposase families like Tn3, IS-*6100*, and integrase core domain were predominantly present in the dumpsite and the 1 km datasets (Table S6). At the dumpsite there was also a notable enrichment of error prone DNA repair genes and genes facilitating enhanced mutation rates. Finally, the dumpsite and the 1 km datasets showed high relative abundance and diversity of proteins involved in transposition and conjugation mechanisms. The overall functional diversity based on KEGG [34] enzyme profiling clearly revealed the impact of HCH and salinity on microbial responses. For instance, the dumpsite and the 5 km datasets had the least correlation (R^2^∶0.92), whereas the dumpsite and 1 km datasets were more correlated (R^2^∶0.943), while 1 km and 5 km datasets were the most correlated (R^2^∶0.98) (Fig. 2D). When the metagenomic data was analyzed at a higher functional category, the contributions of functional genes from eukaryotes was significantly higher at the 5 km, while bacteria contributed more significantly to the metabolic potential of the dumpsite (data not shown).

### Community Potential and Participation in HCH Degradation

**Figure 3:**
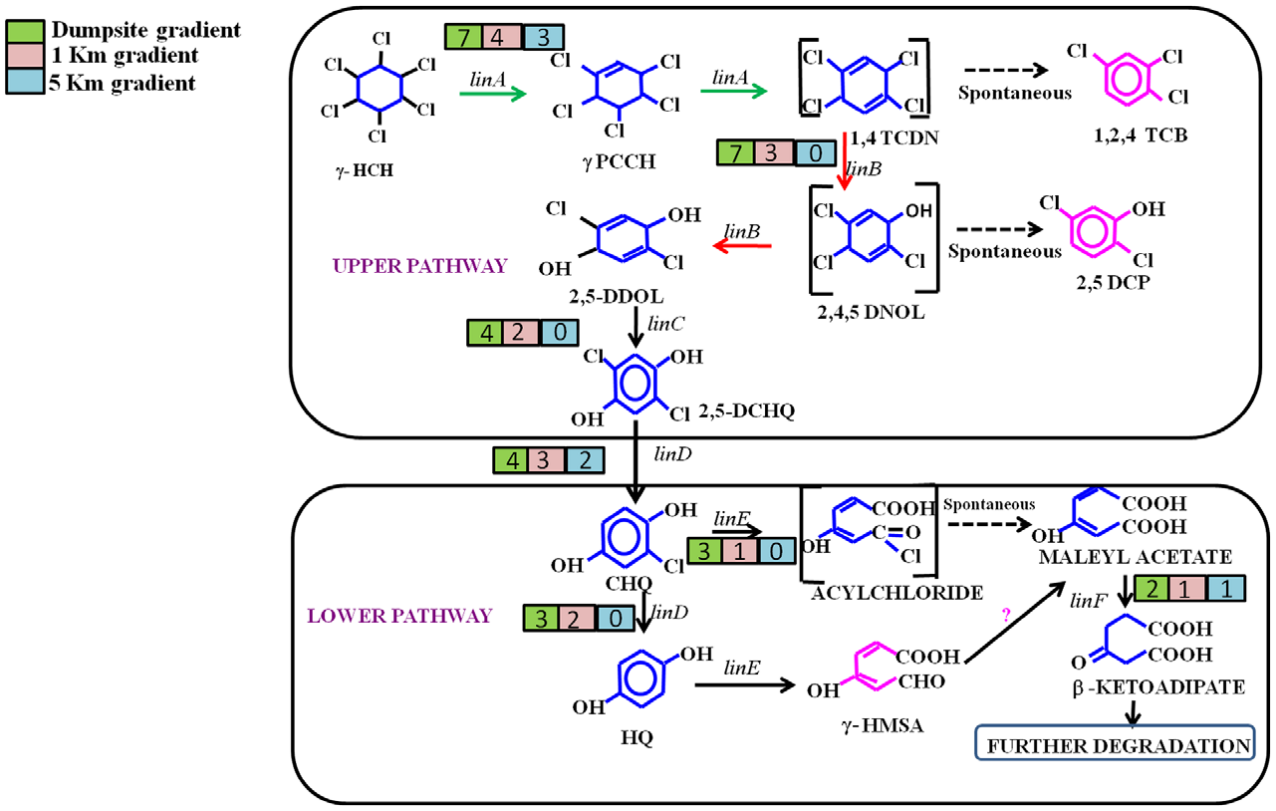
Enrichment of lindane degradation (aerobic) pathway. Schematic representation for the enrichment of aerobic degradation pathway of lindane. Numerical values (on color gradient) at each enzyme represent the diversity (genera) of the corresponding gene present at each metagenome estimated using Transpipe analysis.

To know the relative enrichment of genes already assigned to HCH degradation pathway, functional binning was performed on each of the datasets using BLASTN [19] and transpipe [33] analysis. We were able to bin reads against 12 unique genes that have already been reported to be involved in the HCH degradation pathways. Notable among these are: *linA, linB, linC*, dehydrochlorinase, chlorocatechol 1,2-dioxygenase, 2,4,6-trichlorophenol monooxygenase, 2,6-dichloro-p-hydroquinone 1,2-dioxygenase, and 2,5-dichloro-2,5-cyclohexadiene-1,4-diol, (chloro) muconate-cycloisomerase, LysR family transcriptional regulator (LinR), TRAP-type mannitol/chloroaromatic compound transport system and periplasmic component (*ttg2* gene) (Fig. 3 and Table S7). We compared the three datasets for the presence and relative abundance of HCH degradation genes (*lin* genes). The dumpsite and 1 km site had a higher metabolic potential to degrade HCH isomers, compared to the 5 km site in which these genes were nearly absent (Fig. 3). Additionally, ABC transporter genes like *ttg*2 [64] and Ton-B receptors [65] were found in higher relative abundance at the dumpsite in comparison to the other datasets. These transporter genes have been reported from Sphingomonads where they help in the transport of complex hydrophobic compounds like HCH across the membrane thus facilitating the degradation process [64].

**Figure 4:**
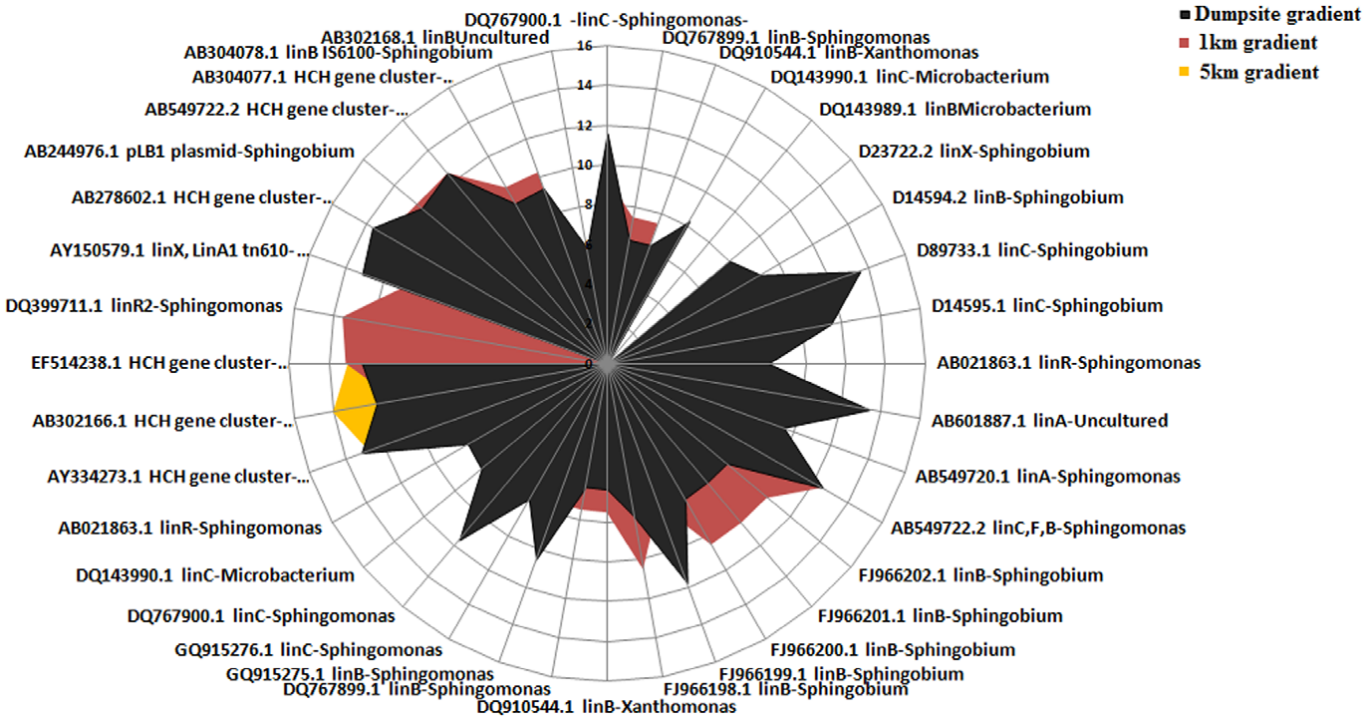
DNA-seq analysis of the community potential for HCH degradation. DNA-seq analysis of metagenome sequences against reference *lin* genes using Array star, x axis represents the relative abundance of *lin* genes from different genera (Table S8) present at studied metagenomes.

Sequences (Table S8) related to the *lin* operon, gene clusters and plasmids were downloaded from NCBI and each of the metagenomes were reference assembled to existing *linA,B,C,D,E,R,X* genes and plasmids. We found 34,953 matches in the dumpsite metagenomic data, 35,256 in the 1 km site, and only 24,442 sequences from the 5 km site. Results from DNA-Seq based analysis (Fig. 4) were in agreement with those of functional binning, HCH contamination levels and taxonomic enrichment studied in each of the metagenomes. We observed a very high relative abundance of genes encoding for Lin A and Lin B, as these two primary enzymes are responsible for the degradation of all HCH isomers and also some of the intermediates (Fig. 3 and 4). We observed that *linA*, *linB*, and *linC* genes were abundant at the dumpsite and 1 km datasets (Fig. 3 and 4) indicating that either a large majority of bacteria contain these genes or that these genes were present in multiple copies as two copies of *linA* gene have already been reported from Sphingomonads that harbor these genes [13], [64]. Our previous studies have revealed certain end products of degradation of α, β and δ HCH under aerobic condition by using *Sphingobium indicum* B90A, and also under anaerobic conditions [5]. However, the enrichment of benzoate, toluene, naphthalene and aromatic ring opening genes at the HCH dumpsite (Table S6) is an indicator that even the end products are degraded further.

### Recruiting *Chromohalobacter Salexigens* Pangenome and Tracing Horizontal Gene Transfer Potential of *lin* Genes *in situ*

Metagenomic studies enable the recovery of partial genetic information from a broad distribution of the community membership. However, for the dominant organism (or pan organism) in a given community it is often possible to reassemble a complete genome, albeit a pan-genome comprised of sequences from a number of closely related species or strains [65]–[66]. Based on the phylogenetic profiles generated by TEFAP, metagenomic SSUs and direct comparison of EGTs to reference genomes, we generated metagenomic recruitment plots for various reference genomes (Table S9) using MUMMER [35]. *De-novo* assembly (see material and methods) of all three datasets resulted into 2,388,526 contigs (N50 = 745 bp, maximum contig size = 3458 bp, average contig coverage = ∼5X). Owing to the primary focus of our further assembly efforts to reconstruct the enriched, salinity tolerant and HCH degrading draft or complete pangenomes (genomic fragments from similar species), *de-novo* assembled contigs were clustered based upon their nucleotide compositional characteristics (tetra nucleotide frequencies and %G+C) as explained earlier [40], [67].

**Figure 5:**
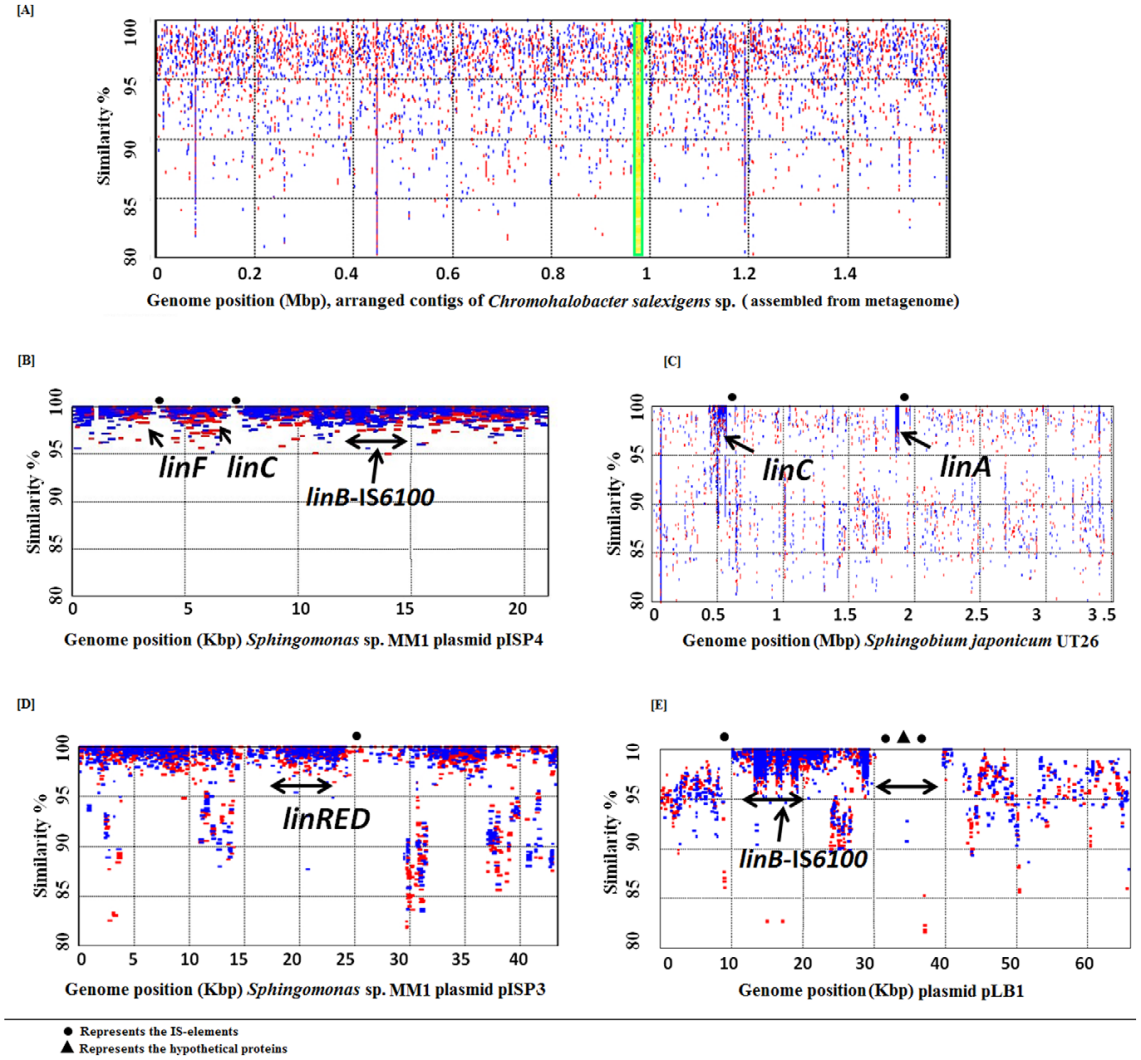
Quantifying the enrichment of environmental genomes/plasmids. Metagenomic recruitment plots of genomes/plasmids constructed using all three studied metagenome sequences. Reads were mapped with coverage parameter. (A) Assembled contigs of *Chromohalobacter salexigens* (5189 contigs) from mtegenomic reads, shaded region represents the location of 16SrRNA gene sequence. (B) pISP4, (C) *Sphingobium japonicum* UT26 chromosome 1, (D) pISP3 and (E) pLB1. Localization of *lin* genes on respective genomes is marked along with representation symbols for IS-elements.

Owing to the relatively high abundance of *Chromohalobacter salexigens* DSM 3043 in our taxonomic analysis, a draft pan-genome of *Chromohalobacter* sp. was constructed from the metagenome data (Fig. 5A, S1, S4). The *Chromohalobacter* sp. assembly consists of 5189 contigs (average contig size = 513 bp, average coverage ∼8X) totaling 1,580 kbp of total draft pan-genome (Fig. 5A and S4). The RAST annotation server [42] was used to annotate 778 protein coding sequences (CDS) and 189 hypothetical proteins on the contigs that were confirmed with an average BLASTp identity of 98.5% to the reference coding sequences.

These observations clearly indicate the enrichment of *Chromohalobacter* over an increasing HCH contamination level, as observed by TEFAP analysis. We were able to assemble the complete 16S rRNA gene sequence of *Chromohalobacter* sp. (99.9% identical to 16S rRNA gene sequence of *Chromohalobacter salexigens* DSM 3043; (Contig no = 646 size = 1652 bp, coverage = >35). Since there was no other 16S rRNA gene sequence (phylogenetic marker) of *Chromohalobacter salexigens* in our assembly it certainly indicates low interstrain microdiversity of *Chromohalobacter salexigens* (average BLASTp identity to the reference coding sequences = 98.5%). It is essential to note that potassium cations released by the pesticide in contaminated soils can lead to an increase in the total salinity of the soil matrix [46]. *Chromohalobacter salexigens* DSM 3043 is a halophilic gamma-proteobacterium with a versatile metabolism allowing fast growth on a large variety of simple carbon compounds as its sole carbon and energy source. This bacterium is also resistant to saturated aromatic hydrocarbons and heavy metals and is a host to several versatile plasmids [68]–[69]. As with other studies that highlight the *in-silico* potential for re-assembled genomes to support specific phenotypes, the role of these organisms in HCH degradation needs to be confirmed through biochemical tests. However, this information could help to refine the culture conditions necessary for axenic isolation in this organism(s), for example by generating a flux balance metabolic model of the organism (e.g. ModelSEED) [70].

The *lin* genes are already known for their mobile nature and association with IS-elements [64] however, there is no evidence of their relative mobility or evolution. Previous reports on the localization of *lin* genes especially *linA*, *linB*, *linC*, *linDER* across different species indicate that many of these genes are present across genomes as well as plasmids [12], [71]–[73]. Recently the presence of *lin* genes has been reported on the genome (3.51 Mbp) of *Sphingobium japonicum* UT26 [12] and plasmids; pISP3 (43k bp) and pISP4 (21k bp) in *Sphingomonas sp*. MM1 [74]. An exogenous plasmid pLB1 (21k bp) that carried IS-*6100* composite transposon containing two copies of *linB* [75] was isolated directly from HCH contaminated soil. Thus we targeted our assembly efforts (clustering using tetra-ESOM and %GC character) to understand the microdiversity and organization of *lin* genes as metagenomic islands using reference sequences of the genome of *Sphingobium japonicum* UT26 (the solitary representative sequenced genome of HCH degrading bacterium available so far) and three plasmids pISP3, pISP4 and pLB1. For this purpose, we generated metagenomic recruitment plots and binned the contigs for the first chromosome of UT26 and three plasmids. Metagenomic recruitment plots of genome and plasmids (Fig. 5. A, B, C, D and E) clearly showed an abundance of metagenomic reads against reference sequences in the range of 97% to 100%. When metagenomic islands were identified over the recruitment plots it became evident that except for the IS-element of the *linB* gene there were hardly any reads mapped over the IS-elements related to the other *lin* genes (Fig. 5. B, C, D and E). This suggested a relative genomic plasticity and faster rate of evolution for various *linA, linC, linDER* and *linF* over *linB* genes. The studies also reflect that the bacterial community at the dumpsite is enriched for HCH degradation potential (*lin* genes), insertion elements, integrases, prophages and/or plasmids, which are contributing in the continuous genetic adaptation of these bacteria.

### Conclusions

This is the first metagenomic analysis of samples collected from soils with differential concentration of HCH contamination. Though the presence of halophilic bacteria can be attributed to strong salinity differences between the dumpsite and the other two sites, the enrichment and diversity of *lin* genes suggests that HCH contamination did play a significant role in structuring the functional potential of the community. This study has shown the enrichment of ubiquitous but yet unknown archaeal, bacterial and fungal taxa under HCH contamination (and highly saline conditions). A higher diversity and abundance of *lin* genes, transposons, plamids, prophages, ABC transporters and genes associated with chemotaxis/motility and membrane transport were observed at the HCH dumpsite dataset. The data thus provided strong evidence not only for the enrichment of a specific microbial population and genes but a massive lateral transfer of catabolic genes (*lin*) through conjugation and transposition among the members of the established microbial community. We recovered one partial enriched microbial genome and three nearly-complete plasmids containing *lin* genes, indicating that these bacteria harbor catabolic plasmids, and dominate this HCH stressed environment. While the results presented here can prove to be an invaluable supplement for the on-going efforts in the development of *in-situ* bioremediation technologies for HCH, this study also suggests good prospects for developing economically viable HCH bioremediation technology. The latter may involve the use of specific tailor- made nutrients(s) and chemicals like taxa specific minimal salt medium [10], and various electron donors [11]. In addition, this study also points out that bioaugmentation by using a consortium (cultivable representatives of the enriched genera) of both HCH degraders and non-degraders could improve the efficiency of remediation efforts that focus on the use of a single taxon.

## Supporting Information

Figure S1 **Two way clustering of bacterial genus (predicted by EGT mapping to NCBI genomes, SSU rRNA analysis against GreenGenes database and by taxa specific 16S rRNA pyrotagging) versus sample matrix.** Genera and sample categories were clustered using Manhattan distance metric, top 50 genera with standard deviation >0.4 and having at least 0.8% of the total abundance were selected. Colour scale is representing the relative abundance of sequence reads after normalising the data from the respective means of individual column (one sample).
(TIF)

Figure S2 **PCA (principle component analysis) performed on the total diversity patterns (phylum) obtained after EGT mapping, metagenomic SSU rRNA analysis and taxa specific pyro-tagging.** Correlation matrix was selected for the co-ordination with 1000 bootstrap values.
(TIF)

Figure S3 **Phylogentic analysis of fungal 18S rRNA gene sequences.** Phylogenetic analysis was performed on the partial (300 bp) 18S rRNA gene sequences obtained from bTEFAP analysis of dumpsite metagenome (n = 42) and reference sequences (n = 49) using the neighbour joining method with Kimura two-parameter model. The bootstrapped consensus tree, inferred from 1,000 replicates is presented as a radial tree. Bootstrap values (percentages of replicate trees in which the associated taxa clustered together) are shown for selected nodes in the tree. The tree is drawn to scale, with branch lengths corresponding to the evolutionary distances used to infer the phylogenetic tree.
(TIF)

Figure S4 **Schematic representation of graft pangenome (contigs) of** ***Chromohalobacter salexgens*** **sp. assembled using tetraESOM and %GC based clustering on de-novo assembled metagenome contigs.** (A) Circular representation of the draft genome (contigs bin). From outside towards the centre: outermost circle, metagenomic contigs arranged using reference sequence, circle 2, metagenomic reads coverage (coordinates with <8X coverage are not represented); circle 3; innermost circle, GC content of the contigs. (B) Contigs are ordered using reference genome sequence (representing by black base ring). Red colored positions represent the non coding tRNA and rRNA genes.
(TIF)

Table S1 **List of specific primers used in the present study for TEFAP (Tag- Encoded FLX Amplicon Pyrosequencing) analysis: First four primer sets in the first column were used for bacterial selective assay.**
(DOCX)

Table S2 **Relative abundance (percentage) of anaerobic bacteria (HCH degradation related) at all three metagenomes obtained after bTEFAP analysis using four bacterial assays.**
(DOCX)

Table S3 **The bacterial genera which were unique to the dumpsite dataset.** The average relative percentage across each of the 4 bacterial diversity assays is presented. For the dumpsite the standard deviation is also provided. For both the one km and 5 km sites each of the assays was negative for these genera.
(DOCX)

Table S4 **Genera enriched in the pristine 5 km compared to the one km dumpsite soil sample.** Those which were significantly higher based upon ANOVA and Tukey-Kramer among the diversity assays are in bold.
(DOCX)

Table S5 **Phylum distributions defined by SSUrRNA typing against Ribosomal Database Project (RDP).** The relative percentage of each bacterial phylum from each site is provided.
(DOCX)

Table S6 **Metagenome annotations at various ranks.** Percentage of total reads mapped to each category is given in respective columns.
(DOCX)

Table S7 **Sequence recruitment for various** ***lin*** **genes (reference sequences).**
(DOCX)

Table S8 **The relative expression based upon an RNA-seq based analysis.** The NCBI sequences for the noted accessions were utilized as the reference transcriptome and the raw reads from each of the 3 metagenomic sites were compared. The genera of the NCBI genes and the gene designations are also indicated.
(DOCX)

Table S9 **List of reference genotypes used in this study to construct metagenomic recruitment plots.**
(DOCX)
