# Supplementary figures and images for: Comparative Metagenomic Analysis of Soil Microbial Communities across Three Hexachlorocyclohexane Contamination Levels
Source: PLoS One. 2012 Sep 28;7(9):e46219. doi: 10.1371/journal.pone.0046219 (PMC3460827; doi:10.1371/journal.pone.0046219)

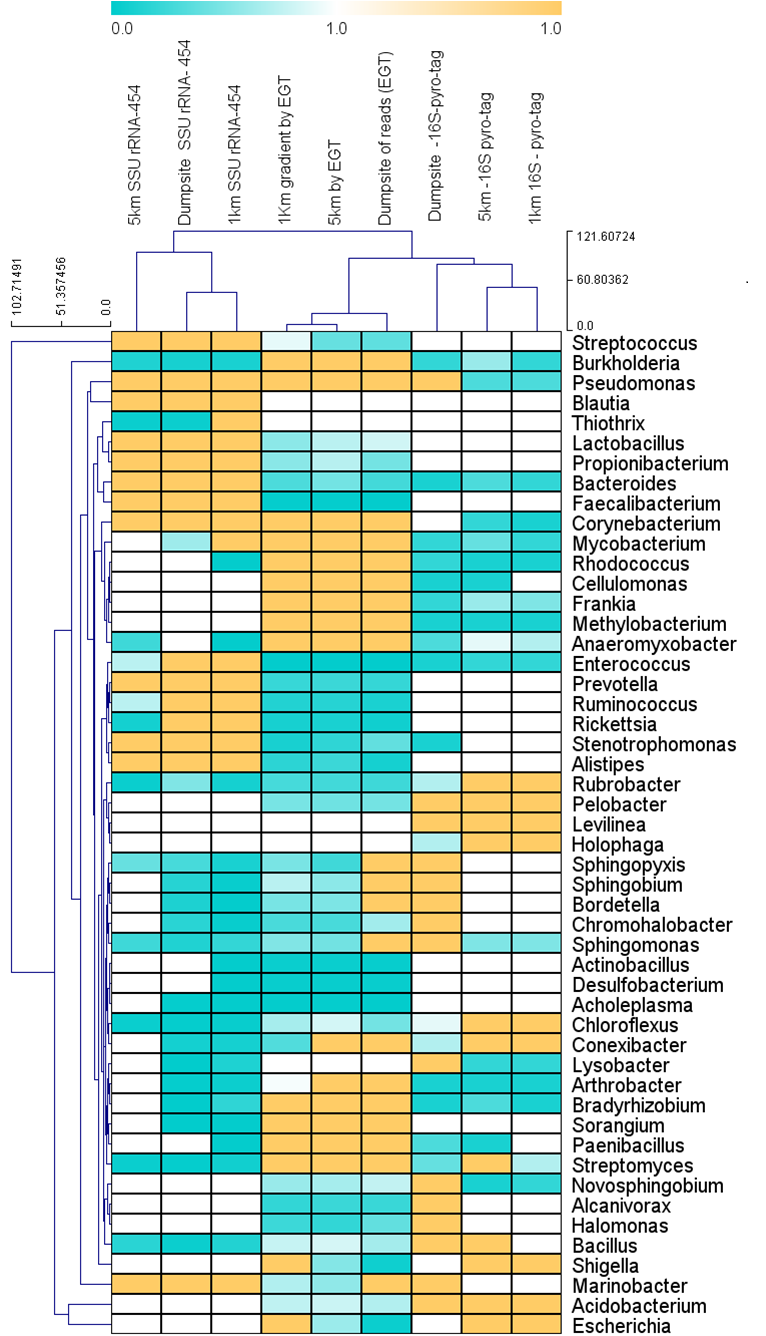

Supplement: Figure S1 — Two way clustering of bacterial genus (predicted by EGT mapping to NCBI genomes, SSU rRNA analysis against GreenGenes database and by taxa specific 16S rRNA pyrotagging) versus sample matrix. Genera and sample categories were clustered using Manhattan distance metric, top 50 genera with standard deviation >0.4 and having at least 0.8% of the total abundance were selected. Colour scale is representing the relative abundance of sequence reads after normalising the data from the respective means of individual column (one sample). (TIF) [file pone.0046219.s001.tif]

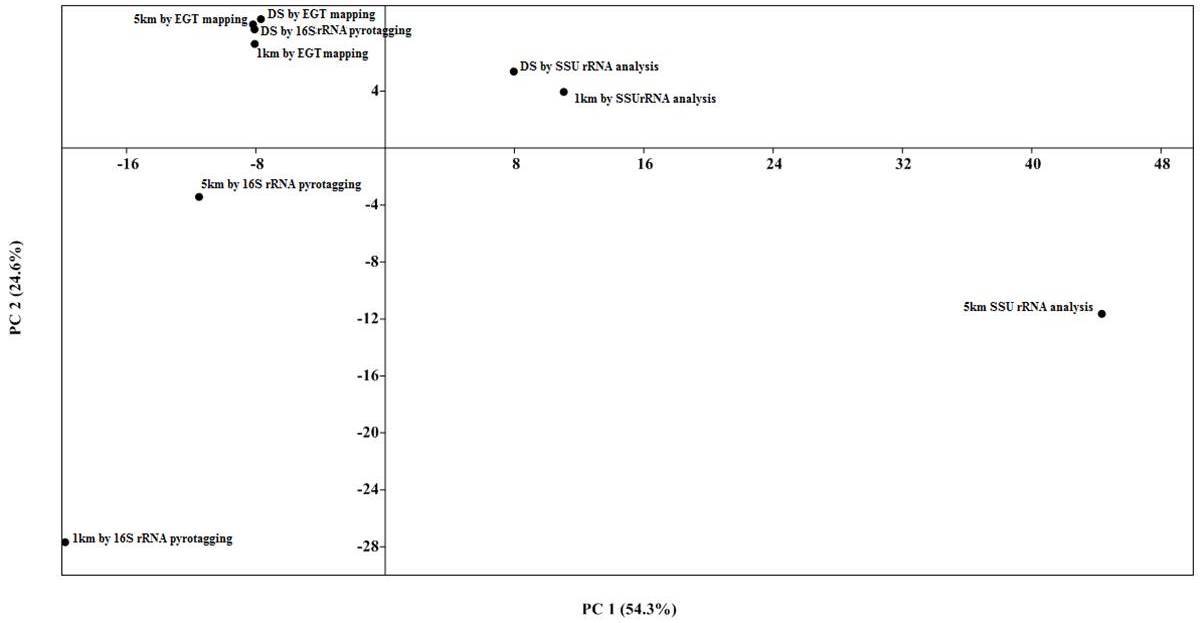

Supplement: Figure S2 — PCA (principle component analysis) performed on the total diversity patterns (phylum) obtained after EGT mapping, metagenomic SSU rRNA analysis and taxa specific pyro-tagging. Correlation matrix was selected for the co-ordination with 1000 bootstrap values. (TIF) [file pone.0046219.s002.tif]

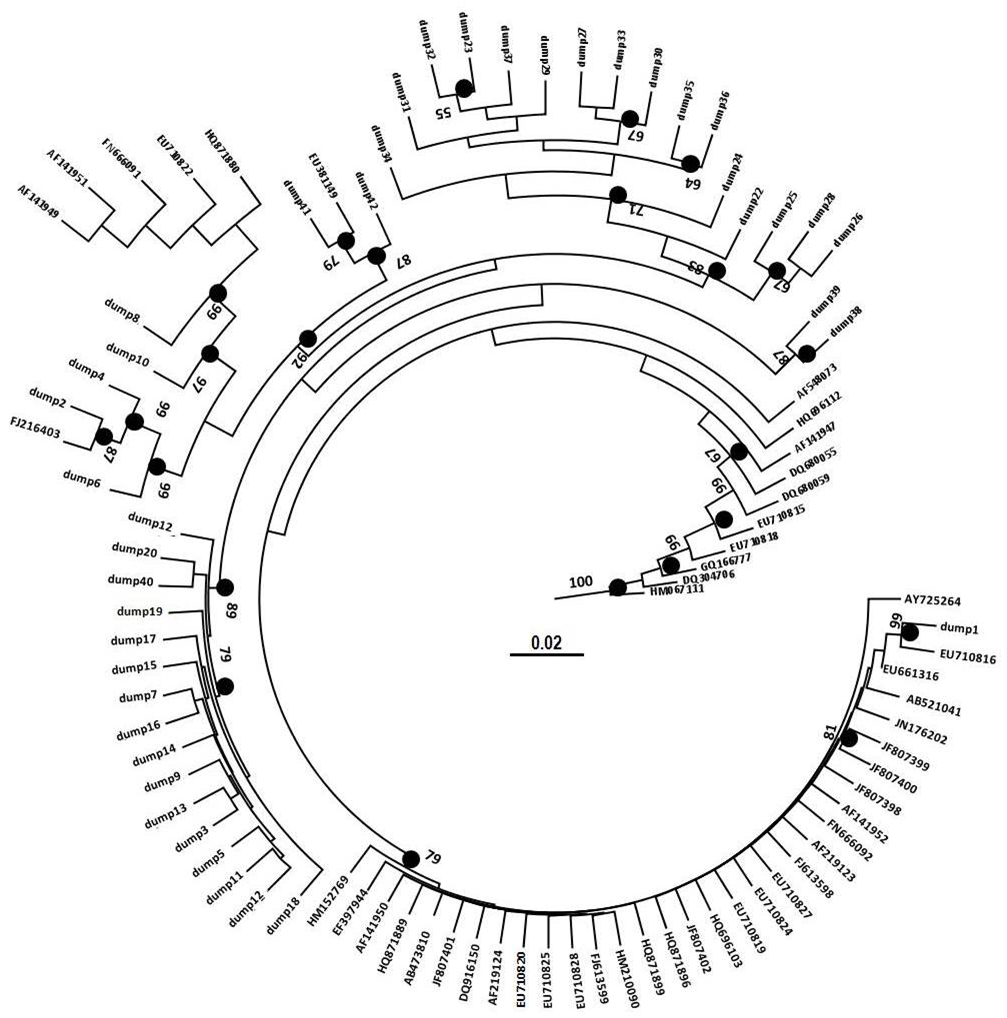

Supplement: Figure S3 — Phylogentic analysis of fungal 18S rRNA gene sequences. Phylogenetic analysis was performed on the partial (300 bp) 18S rRNA gene sequences obtained from bTEFAP analysis of dumpsite metagenome (n = 42) and reference sequences (n = 49) using the neighbour joining method with Kimura two-parameter model. The bootstrapped consensus tree, inferred from 1,000 replicates is presented as a radial tree. Bootstrap values (percentages of replicate trees in which the associated taxa clustered together) are shown for selected nodes in the tree. The tree is drawn to scale, with branch lengths corresponding to the evolutionary distances used to infer the phylogenetic tree. (TIF) [file pone.0046219.s003.tif]

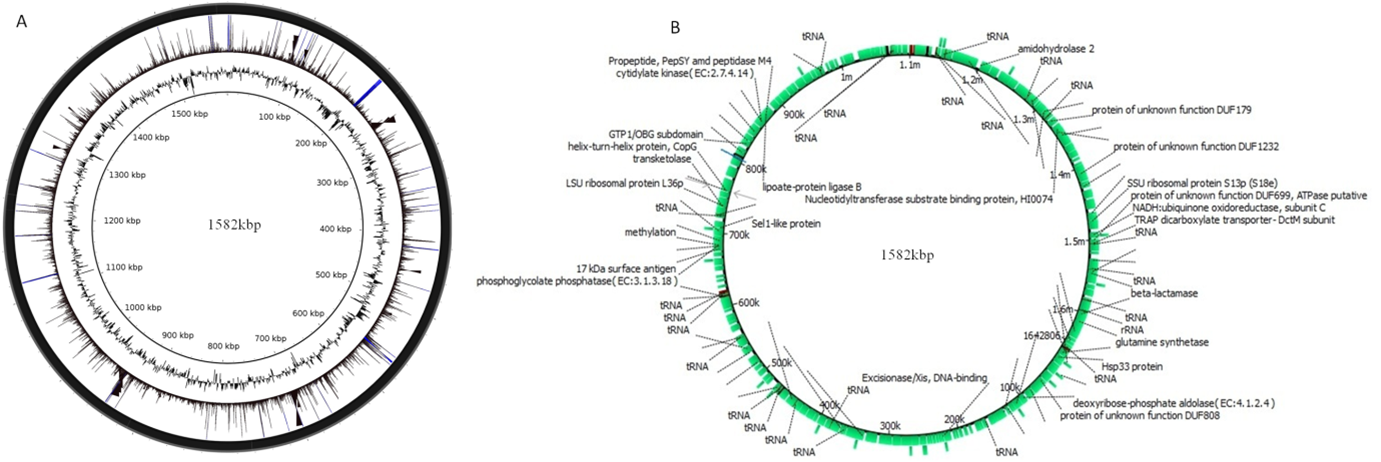

Supplement: Figure S4 — Schematic representation of graft pangenome (contigs) of Chromohalobacter salexgens sp. assembled using tetraESOM and %GC based clustering on de-novo assembled metagenome contigs. (A) Circular representation of the draft genome (contigs bin). From outside towards the centre: outermost circle, metagenomic contigs arranged using reference sequence, circle 2, metagenomic reads coverage (coordinates with <8X coverage are not represented); circle 3; innermost circle, GC content of the contigs. (B) Contigs are ordered using reference genome sequence (representing by black base ring). Red colored positions represent the non coding tRNA and rRNA genes. (TIF) [file pone.0046219.s004.tif]
