# Supplementary material for: Comparative Metagenomic Analysis of Soil Microbial Communities across Three Hexachlorocyclohexane Contamination Levels
Source: PLoS One. 2012 Sep 28;7(9):e46219. doi: 10.1371/journal.pone.0046219 (PMC3460827; doi:10.1371/journal.pone.0046219)
Supplement: Table S1 — List of specific primers used in the present study for TEFAP (Tag- Encoded FLX Amplicon Pyrosequencing) analysis: First four primer sets in the first column were used for bacterial selective assay. (DOCX) [file pone.0046219.s005.docx]

| Primer sets | Forward 5’-3’ | Reverse 5’–3’ |
| --- | --- | --- |
| 28F-519R | TTTGATCNTGGCTCAG | GWNTTACNGCGGCKGCTG |
| 939F-1492R | TTGACGGGGGCCCGCAC | TACCTTGTTACGACTT |
| 530F-1100R | GTGCCAGCMGCNGCGG | GGGTTNCGNTCGTTR |
| 515F-806R | GTGCCAGCMGCCGCGGTAA | GGACTACHVGGGTWTCTAAT |
| Archaea selective | GYGCASCAGKCGMGAAW | GGACTACVSGGGTATCTAAT |
| Fungal selective SSU | TGGAGGGCAAGTCTGGTG | TCGGCATAGTTTATGGTTAAG |
