# Supplementary material for: Comparative Metagenomic Analysis of Soil Microbial Communities across Three Hexachlorocyclohexane Contamination Levels
Source: PLoS One. 2012 Sep 28;7(9):e46219. doi: 10.1371/journal.pone.0046219 (PMC3460827; doi:10.1371/journal.pone.0046219)
Supplement: Table S2 — Relative abundance (percentage) of anaerobic bacteria (HCH degradation related) at all three metagenomes obtained after bTEFAP analysis using four bacterial assays. (DOCX) [file pone.0046219.s006.docx]

| Genera Name | Dumpsite | One km | 5 km |
| --- | --- | --- | --- |
| *Clostridium* | 0.94 | 0.44 | 0.77 |
| *Citrobacter* | 0.0060 | 0.0225 | 0.0093 |
| *Desulfovibrio* | 0.024 | 0.040 | 0.0696 |
| *Desulfococcus* | 0 | 0.007 | 0 |
| *Dehalobacter* | 0.0042 | 0 | 0 |
