# Supplementary material for: Comparative Metagenomic Analysis of Soil Microbial Communities across Three Hexachlorocyclohexane Contamination Levels
Source: PLoS One. 2012 Sep 28;7(9):e46219. doi: 10.1371/journal.pone.0046219 (PMC3460827; doi:10.1371/journal.pone.0046219)
Supplement: Table S3 — The bacterial genera which were unique to the dumpsite dataset. The average relative percentage across each of the 4 bacterial diversity assays is presented. For the dumpsite the standard deviation is also provided. For both the one km and 5 km sites each of the assays was negative for these genera. (DOCX) [file pone.0046219.s007.docx]

| Genera Name | Avg 5 Km | Avg one Km | Avg Dumpsite | Std. Dev Dumpsite |
| --- | --- | --- | --- | --- |
| *Marinimicrobium* | 0 | 0 | 1.12 | 0.45 |
| *Idiomarina* | 0 | 0 | 0.67 | 0.16 |
| *Salinisphaera* | 0 | 0 | 0.46 | 0.20 |
| *Pelagibius* | 0 | 0 | 0.23 | 0.15 |
| *Microbulbifer* | 0 | 0 | 0.16 | 0.12 |
| *Acidisoma* | 0 | 0 | 0.09 | 0.09 |
| *Ochrobactrum* | 0 | 0 | 0.09 | 0.08 |
| *Arhodomonas* | 0 | 0 | 0.08 | 0.05 |
| *Pullulanibacillus* | 0 | 0 | 0.07 | 0.01 |
| *Salegentibacter* | 0 | 0 | 0.05 | 0.02 |
| *Methylonatrum* | 0 | 0 | 0.04 | 0.06 |
| *Virgibacillus* | 0 | 0 | 0.03 | 0.01 |
