# Supplementary material for: Comparative Metagenomic Analysis of Soil Microbial Communities across Three Hexachlorocyclohexane Contamination Levels
Source: PLoS One. 2012 Sep 28;7(9):e46219. doi: 10.1371/journal.pone.0046219 (PMC3460827; doi:10.1371/journal.pone.0046219)
Supplement: Table S4 — Genera enriched in the pristine 5 km compared to the one km dumpsite soil sample. Those which were significantly higher based upon ANOVA and Tukey-Kramer among the diversity assays are in bold. (DOCX) [file pone.0046219.s008.docx]

| Genera Name | 5km | 1km | Dumpsite |
| --- | --- | --- | --- |
| *Acidobacterium* | 17.32 | 8.60 | 7.02 |
| *Levilinea* | 3.50 | 2.14 | 1.21 |
| *Rubrobacter* | 3.30 | 1.89 | 0.67 |
| *Pelobacter* | 3.12 | 1.57 | 1.17 |
| *Holophaga* | 2.53 | 1.62 | 0.69 |
| ***Caldilinea*** | 2.16 | 1.00 | 0.80 |
| *Conexibacter* | 1.99 | 1.34 | 0.69 |
| *Chloroflexus* | 1.65 | 1.10 | 0.86 |
| ***Streptomyces*** | 1.16 | 0.69 | 0.43 |
| *Verrucomicrobium* | 1.01 | 0.82 | 0.20 |
| ***Gemmatimonas*** | 1.00 | 0.46 | 0.37 |
| *Solirubrobacter* | 0.99 | 0.65 | 0.43 |
| ***Byssovorax*** | 0.97 | 0.56 | 0.30 |
| *Anaeromyxobacter* | 0.86 | 0.74 | 0.31 |
| ***Geobacter*** | 0.81 | 0.67 | 0.22 |
| *Rhodopseudomonas* | 0.70 | 0.20 | 0.04 |
| ***OP10 (genus)*** | 0.70 | 0.51 | 0.20 |
| *Solibacter* | 0.67 | 0.54 | 0.40 |
| *Thiobacillus* | 0.66 | 0.46 | 0.18 |
| *Frankia* | 0.61 | 0.49 | 0.20 |
| *Burkholderia* | 0.57 | 0.23 | 0.23 |
| ***Plesiocystis*** | 0.03 | 0.00 | 0.00 |
| ***Pedomicrobium*** | 0.16 | 0.02 | 0.01 |
| ***Adhaeribacter*** | 0.11 | 0.06 | 0.01 |
