# Supplementary material for: Comparative Metagenomic Analysis of Soil Microbial Communities across Three Hexachlorocyclohexane Contamination Levels
Source: PLoS One. 2012 Sep 28;7(9):e46219. doi: 10.1371/journal.pone.0046219 (PMC3460827; doi:10.1371/journal.pone.0046219)
Supplement: Table S5 — Phylum distributions defined by SSUrRNA typing against Ribosomal Database Project (RDP). The relative percentage of each bacterial phylum from each site is provided. (DOCX) [file pone.0046219.s009.docx]

| Name | 1 Km | 5Km | Dumpsite |
| --- | --- | --- | --- |
| Proteobacteria | 50.8 | 27.0 | 50.0 |
| Firmicutes | 33.8 | 70.0 | 43.0 |
| Actinobacteria | 14.5 | 2.2 | 4.0 |
| Bacteroidetes | 0.2 | 0.5 | 2.7 |
| Cyanobacteria | 0.0 | 0.2 | 1.0 |
| Fusobacteria | 0.0 | 0.1 | 0.0 |
| Chlorobi | 0.5 | 0.1 | 0.0 |
| Spirochaetes | 0.2 | 0.0 | 0.1 |
| Tenericutes | 0.0 | 0.0 | 0.0 |
