# Supplementary material for: Comparative Metagenomic Analysis of Soil Microbial Communities across Three Hexachlorocyclohexane Contamination Levels
Source: PLoS One. 2012 Sep 28;7(9):e46219. doi: 10.1371/journal.pone.0046219 (PMC3460827; doi:10.1371/journal.pone.0046219)
Supplement: Table S6 — Metagenome annotations at various ranks. Percentage of total reads mapped to each category is given in respective columns. (DOCX) [file pone.0046219.s010.docx]

| **Cellular processes** | **Subsystem** | **Protein family** | **Dumpsite** | **1km** | **5km** |
| --- | --- | --- | --- | --- | --- |
| **Cellular processes** | **Subsystem** | **Protein family** | **Dumpsite** | **1km** | **5km** |
| Membrane transport |  | ABC transporter | 1.9 | 1.6 | 1.3 |
|  |  | Inner-membrane tranporter | 0.09 | 0.03 | 0.05 |
|  |  | Multidrug efflux protein | 0.83 | 0.59 | 0.003 |
|  |  | Na+ transporter | 0.012 | 0.003 | 0.0026 |
|  |  | Proton dependent transporter | 0.006 | 0.004 | 0.0029 |
|  |  |  |  |  |  |
| Motility and chemotaxis |  |  |  |  |  |
|  | Flagellar activity | Flagellar motility regulators | 0.002 | 0.004 | 0.0016 |
|  |  | Flagelar basal body protein FlaE | 0.006 | 0.00024 | 0.0032 |
|  |  | Flagellar protein Flis | 0.007 | 0.005 | 0.002 |
|  |  | Flagellar protein Flit | 0.005 | 0.006 | 0.001 |
|  |  | Flagellar hook basal body protein | 0.009 | 0.004 | 0.002 |
|  |  |  |  |  |  |
| Phages, Prophages and |  |  |  |  |  |
| plasmid encoded functions |  |  |  |  |  |
|  | Transposition | Transposase | 0.563 | 0.37 | 0.138 |
|  |  | IS family | 0.213 | 0.045 | 0.029 |
|  |  | IS2100 | 0.08 | 0.01 | 0.009 |
|  |  | phage integrases | 0.032 | 0.037 | 0.014 |
|  |  | Mu Transposase C-terminal | 0.0004 | 0.003 | 0.001 |
|  |  | Transposase domain DUF772 | 0.44 | 0.42 | 0.025 |
|  |  | IS116/IS110/IS902 family | 0.58 | 0.31 | 0.029 |
|  |  | Tnp2 | 0.003 | 0.0041 | 0.044 |
|  |  | IS1 | 0.07 | 0.05 | 0.0027 |
|  |  | Mu-transposase | 0.004 | 0.002 | 0.019 |
|  |  | Bacillus transposase | 0.002 | 0.0015 | 0 |
|  |  |  |  |  |  |
| Metabolism of Aromatic |  |  |  |  |  |
| compounds | Benzoate degradtion |  | 0.89 | 0.03 | 0.019 |
|  | toluene degradation |  | 0.03 | 0.0028 | 0 |
|  | homogentisate degradation | | 0.005 | 0.003 | 0.0001 |
|  | napthalene degradation | | 0.04 | 0.003 | 0 |
|  | lignin gegradation | aromatic ring clevage | 0.003 | 0.0012 | 0.0019 |
