# Supplementary material for: Comparative Metagenomic Analysis of Soil Microbial Communities across Three Hexachlorocyclohexane Contamination Levels
Source: PLoS One. 2012 Sep 28;7(9):e46219. doi: 10.1371/journal.pone.0046219 (PMC3460827; doi:10.1371/journal.pone.0046219)
Supplement: Table S7 — Sequence recruitment for various lin genes (reference sequences). (DOCX) [file pone.0046219.s011.docx]

| Gene name | Reference | %Similarity | Sequences in DS, 1km, 5km |
| --- | --- | --- | --- |
| *LinA*1 (Dehydrochlorinase) | *Sphingobium indicum* B90A | 80-99 | 87, 62, 3 |
| *LinA2* (Dehydrochlorinase) | *Sphingobium indicum* B90A | 80-99 | 111, 71, 9 |
| *LinB* (Haloalkane dehalogenase) | *Sphingobium japonicum* UT 26 | 80-97 | 231, 148, 11 |
| *LinC* (Dehydrogenase) | *Sphingobium japonicum* UT 26 | 84-95 | 235, 49, 9 |
| *LinD* (Reductive dechlorinase) | *Sphingobium japonicum* UT 26 | 88-94 | 154, 83, 2 |
| *LinE* (Dioxygenase) | *Sphingobium japonicum* UT 26 | 89-94 | 138, 92, 5 |
| *LinF* (Maleylacetate reductase) | *Sphingobium japonicum* UT 26 | 95-99 | 103, 36, 14 |
| *LinX* (Dehydrogenases) | *Sphingobium japonicum* UT 26 | 90-97 | 31, 11,0 |

ABREVATION: DS = Dumpsite
