# Supplementary material for: Comparative Metagenomic Analysis of Soil Microbial Communities across Three Hexachlorocyclohexane Contamination Levels
Source: PLoS One. 2012 Sep 28;7(9):e46219. doi: 10.1371/journal.pone.0046219 (PMC3460827; doi:10.1371/journal.pone.0046219)
Supplement: Table S8 — The relative expression based upon an RNA-seq based analysis. The NCBI sequences for the noted accessions were utilized as the reference transcriptome and the raw reads from each of the 3 metagenomic sites were compared. The genera of the NCBI genes and the gene designations are also indicated. (DOCX) [file pone.0046219.s012.docx]

| S.no | NCBI accession (gene) | Related genera |
| --- | --- | --- |
| 1 | DQ767900.1 linC | *Sphingomonas* |
| 2 | DQ767899.1 linB | *Sphingomonas* |
| 3 | DQ910544.1 linB | *Xanthomonas* |
| 4 | DQ143990.1 linC | *Microbacterium* |
| 5 | DQ143989.1 linB | *Microbacterium* |
| 6 | D23722.2 linX | *Sphingobium* |
| 7 | D14594.2 linB | *Sphingobium* |
| 8 | D89733.1 linD | *Sphingobium* |
| 9 | D14595.1 linC | *Sphingobium* |
| 10 | AB021863.1 linR | *Sphingomonas* |
| 11 | AB601887.1 linA | Uncultured |
| 12 | AB549720.1 linA | *Sphingomonas* |
| 13 | AB549722.2 linC,F,B | *Sphingomonas* |
| 14 | FJ966202.1 linB | *Sphingobium* |
| 15 | FJ966201.1 linB | *Sphingobium* |
| 16 | FJ966200.1 linB | *Sphingobium* |
| 17 | FJ966199.1 linB | *Sphingobium* |
| 18 | FJ966198.1 linB | *Sphingobium* |
| 19 | DQ910544.1 linB | *Xanthomonas* |
| 20 | DQ767899.1 linB | *Sphingomonas* |
| 21 | GQ915275.1 linB | *Sphingomonas* |
| 22 | GQ915276.1 linC | *Sphingomonas* |
| 23 | DQ767900.1 linC | *Sphingomonas* |
| 24 | DQ143990.1 linC | *Microbacterium* |
| 25 | AB021863.1 linR | *Sphingomonas* |
| 26 | AY334273.1 HCH gene cluster | *Sphingomonas* |
| 27 | AB302166.1 HCH gene cluster | Uncultured |
| 28 | EF514238.1 HCH gene cluster | *Pseudomonas* |
| 29 | DQ399711.1 linR2 | *Sphingomonas* |
| 30 | AY150579.1 linX, LinA1 tn610 | *Sphingomonas* |
| 31 | AB278602.1 HCH gene cluster | *Sphingobium* |
| 32 | AB244976.1 pLB1 plasmid | *Sphingobium* |
| 33 | AB549722.2 HCH gene cluster | *Sphingomonas* |
| 34 | AB304077.1 HCH gene cluster | *Sphingobium* |
| 35 | AB304078.1 linB IS6100 | *Sphingobium* |
| 36 | AB302168.1 linB | Uncultured |
