# Supplementary material for: Comparative Metagenomic Analysis of Soil Microbial Communities across Three Hexachlorocyclohexane Contamination Levels
Source: PLoS One. 2012 Sep 28;7(9):e46219. doi: 10.1371/journal.pone.0046219 (PMC3460827; doi:10.1371/journal.pone.0046219)
Supplement: Table S9 — List of reference genotypes used in this study to construct metagenomic recruitment plots. (DOCX) [file pone.0046219.s013.docx]

| S.no | Reference Genotype | Accession Number | Total size (Mbp) |
| --- | --- | --- | --- |
| 1 | [*Escherichia coli str. K-12 substr*. W3110](http://www.ncbi.nlm.nih.gov/nuccore/NC_007779.1) | NC_007779 | 4.6 |
| 2 | [*Marinobacter adhaerens* HP15 chromosome](http://www.ncbi.nlm.nih.gov/nuccore/NC_017506.1) | NC_017506 | 4.4 |
| 3 | [*Halomonas elongata* DSM 2581](http://www.ncbi.nlm.nih.gov/nuccore/NC_014532.1) | NC_014532 | 4.06 |
| 4 | [*Pseudomonas aeruginosa* PAO1 chromosome](http://www.ncbi.nlm.nih.gov/nuccore/NC_002516.2) | NC_002516 | 6.2 |
| 5 | [*Novosphingobium aromaticivorans* DSM 12444 chromosome](http://www.ncbi.nlm.nih.gov/nuccore/NC_007794.1) | NC_007794 | 3.5 |
| 6 | [*Novosphingobium* sp. PP1Y chromosome](http://www.ncbi.nlm.nih.gov/nuccore/NC_015580.1) | NC_015580 | 3.9 |
| 7 | [*Sphingomonas wittichii* RW1 chromosome](http://www.ncbi.nlm.nih.gov/nuccore/NC_009511.1) | NC_009511 | 5.3 |
| 8 | [*Alcanivorax borkumensis* SK2 chromosome](http://www.ncbi.nlm.nih.gov/nuccore/NC_008260.1) | NC_008260 | 3.1 |
| 9 | [*Sphingopyxis alaskensis* RB2256 chromosome](http://www.ncbi.nlm.nih.gov/nuccore/NC_008048.1) | NC_008048 | 3.3 |
| 10 | [*Chromohalobacter salexigens* DSM 3043](http://www.ncbi.nlm.nih.gov/nuccore/NC_007963.1) | NC_007963 | 3.6 |
| 12 | [*Sphingobium chlorophenolicum* L-1 chromosome 1](http://www.ncbi.nlm.nih.gov/nuccore/NC_015593.1) | NC_015593 | 3.0 |
| 13 | [*Sphingobium* sp. SYK-6 chromosome](http://www.ncbi.nlm.nih.gov/nuccore/NC_015976.1) | NC_015976 | 4.1 |
| 14 | [*Sphingobium japonicum* UT26S chromosome 1](http://www.ncbi.nlm.nih.gov/nuccore/NC_014006.1) | NC_014006 | 3.5 |
| 15 | [*Sphingobium japonicum* UT26S DNA, chromosome 2](http://www.ncbi.nlm.nih.gov/nuccore/AP010804.1) | AP010804 | 0.681 |
| 16 | [*Sphingobium japonicum* UT26S plasmid pCHQ1](http://www.ncbi.nlm.nih.gov/nuccore/NC_014007.1) | NC_014007 | 0.190 |
| 17 | [*Sphingobium japonicum* UT26S plasmid pUT1 DNA](http://www.ncbi.nlm.nih.gov/nuccore/AP010806.1) | AP010806 | 0.031 |
| 18 | [*Sphingobium japonicum* UT26S plasmid pUT2, complete sequence](http://www.ncbi.nlm.nih.gov/nuccore/NC_014009.1) | NC_014009. | 0.005 |
| 19 | [*Sphingomonas* sp. MM-1 plasmid pISP3](http://www.ncbi.nlm.nih.gov/nuccore/NC_013970.1) | NC_013970 | 0.043 |
| 20 | [*Sphingomonas sp*. MM-1 plasmid pISP4](http://www.ncbi.nlm.nih.gov/nuccore/AB549722.2) | AB549722 | 0.021 |
| 21 | [*Sphingobium sp*. SYK-6 plasmid pSLPG](http://www.ncbi.nlm.nih.gov/nuccore/NC_015974.1) | NC_015974 | 0.148 |
| 22 | [Plasmid pLB1](http://www.ncbi.nlm.nih.gov/nuccore/AB244976.1) | AB244976 | 0.065 |
